# Stem Cell Niche Microenvironment: Review

**DOI:** 10.3390/bioengineering8080108

**Published:** 2021-07-28

**Authors:** Mohamed Abdul-Al, George Kumi Kyeremeh, Morvarid Saeinasab, Saeed Heidari Keshel, Farshid Sefat

**Affiliations:** 1 Department of Biomedical and Electronics Engineering, School of Engineering, University of Bradford, Bradford BD71DP, UK; m.abdul-al@bradford.ac.uk (M.A.-A.); g.k.kyeremeh@bradford.ac.uk (G.K.K.); 2 Department of Biology, Faculty of Science, Ferdowsi University of Mashhad, Mashhad 91779 48974, Iran; m.saeinasab@gmail.com; 3 Department of Tissue Engineering and Applied Cell Sciences, School of Advanced Technologies in Medicine, Shahid Beheshti University of Medical Sciences, Tehran 19839 69411, Iran; saeedhey@gmail.com; 4 Interdisciplinary Research Centre in Polymer Science & Technology (Polymer IRC), University of Bradford, Bradford BD71DP, UK

**Keywords:** stem cell, niches, microenvironment, cornea

## Abstract

The cornea comprises a pool of self-regenerating epithelial cells that are crucial to preserving clarity and visibility. Limbal epithelial stem cells (LESCs), which live in a specialized stem cell niche (SCN), are crucial for the survival of the human corneal epithelium. They live at the bottom of the limbal crypts, in a physically enclosed microenvironment with a number of neighboring niche cells. Scientists also simplified features of these diverse microenvironments for more analysis in situ by designing and recreating features of different SCNs. Recent methods for regenerating the corneal epithelium after serious trauma, including burns and allergic assaults, focus mainly on regenerating the LESCs. Mesenchymal stem cells, which can transform into self-renewing and skeletal tissues, hold immense interest for tissue engineering and innovative medicinal exploration. This review summarizes all types of LESCs, identity and location of the human epithelial stem cells (HESCs), reconstruction of LSCN and artificial stem cells for self-renewal.

## 1. Introduction

The cornea is the outermost section of the eye surface that allows light to enter and reach the ocular epithelium and activate the neural impulses of the retina [1]. The cornea also serves as a protective boundary between the outer world and the body’s internal systems, shielding the eye from external harm [2].

The cornea is made up of nonkeratinizing squamous epithelium, avascular, collagen-rich epithelial cells that are formed by self-renewing, stratified tissues [3]. The cornea’s transparency is crucial and primarily due to unique characteristics of the corneal stroma. The lack of blood vessels, the distinct organization of collagen fibers and the low percentage of stromal cells are all essential features in this regard [4]. The corneal epithelium lines the stromal surface and defends it from chemical insults. It is also important for the preservation of the stroma’s transparency-enabling properties. Moreover, with the exception of keratinizing epithelia, such as the epidermis, which replaces the cytoplasm of the outer layers with keratin proteins, the corneal epithelium keeps live cells at the edge layer, enhancing transparency.

Considering the ocular epithelium’s barrier structure and the variety of threats it faces, long-term preservation is important, and it is regulated by ESCs (epithelial stem cells) that live within the tissue. Scientists’ understanding of how CESCs (corneal epithelial stem cells) are controlled through disease, homeostasis and recovery is currently inadequate, and further elaboration of the molecular and cellular pathways that govern CESC activity would have significant clinical consequences.

The epithelium layer of the cornea acts as a protective and defensive shield, while also contributing to corneal openness. It is constantly switched over when the most superficial cells of the ocular epithelium fade away and are replaced by limbal epithelial stem cells (LESCs). LESCs are initiated from the limbus region, which is the border between the conjunctiva and cornea [5]. LESCs rely on their unique microenvironment, identified as the limbal niche, for separation, growth and movement. Cells such as mesenchymal cells, nerve cells, melanocytes, skin cells and vascular cells, ECM (extracellular matrix) and signaling molecules distinguish the limbal niche [6,7,8,9,10,11]. Pathology affecting any part of the limbal niche can cause LESC disorder, which leads to successful LSCD (limbal stem cell deficiency) [9,12,13].

The recent understanding of CESC biology has a special emphasis on the development of the stromal microenvironment, or niche, reconstruction of the LSCN (limbal stem cell niche), ASCN (artificial stem cell niche) and in regulating stem cell function.

Limbal biopsy-derived stromal cells (LBSCs) are also important cells within the human eye which can grow very fast. These are highly clonogenic and could generate spheres expressing stem cell genes including Oct4, Nestin, NGFR, PAX6, ABCG2 and Sox2. Human LBSCs can be differentiated into keratocytes expressing characteristic marker genes, including AQP1, ALDH3A1, KERA and PTGDS. LBSCs also exhibit stem cell-like properties, and mesenchymal cells isolated from limbal biopsies were highly clonogenic, irrespective of the culture conditions. LBSCs can also differentiate into keratocytes in vitro. A good example is LBSCs which have been cultured on a collagen gel in ascorbate-containing, serum-free keratocyte differentiation medium (KDM), which resulted as decreased in the expression of the stem cell genes Nestin and *ABCG2,* as well as an increase in expression of some genes which are associated with keratocyte differentiation. LBSCs also promote regeneration of native stromal tissue during wound repair and have been shown to reduce corneal vascularization in mice [14].

Human corneal stromal stem cells (CSSCs) undergo massive expansion in vitro without loss of the ability to adopt a keratocyte phenotype. Several research papers suggested CSSCs to be of neural crest lineage and not from bone marrow. CSSCs are localized in the anterior peripheral (limbal) stroma near stem cells of the corneal epithelium [15]. 

The vasculature of the limbus supplies the peripheral cornea, conjunctiva, epi-sclera, limbal sclera and peripheral uvea [16]. The concept of stem cell niche to act as a unique microenvironment to support self-renewal and multipotential activity was first proposed in the late 1970s [17]. Niches are 3D stem cell-sheltering, highly organized interactive structural units which commonly happen at tissue intersections or transition zones [18]. Molecular crosstalk from surrounding cells and soluble signals from the immediate vasculature or from extracellular matrix (ECM)-sequestered mediators unique to the microenvironment are thought to provide the differential cues that dictate SC homeostatic or activation programs [19]. In this paper, we discuss the importance of stem cell niches in the human eye. 

## 2. Human Cornea

**Figure 1:**
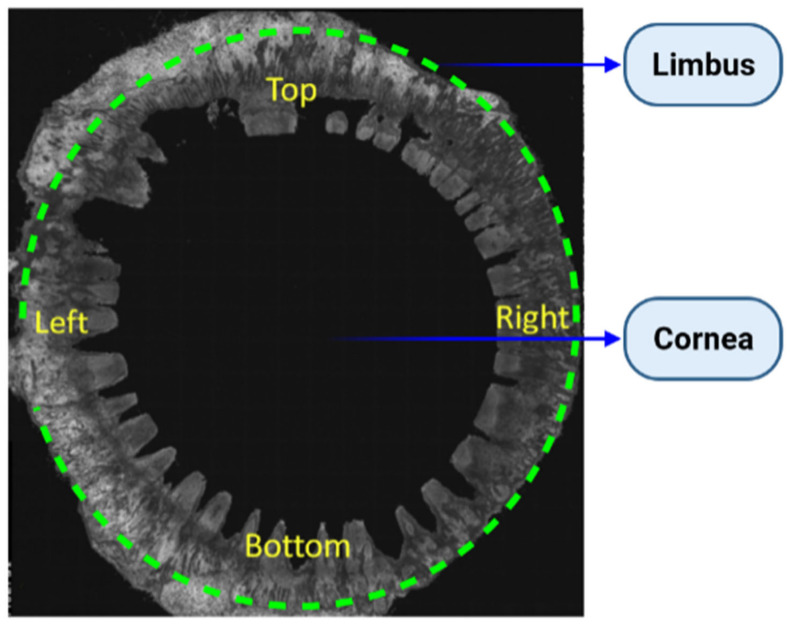
The morphology of the human limbal region for the entire eye. Entire 360° corneoscleral rim, where corneal button has been removed for keratoplasty. Image captured by OCT [6].

The cornea defines the anterior layer of the eye, which is differentiated from the adjacent conjunctiva by a transitional region called the limbus, as shown in Figure 1 [20]. The human cornea is one of the last optimization methods to mature throughout growth. Connections between the overlying surface ectoderm and the lens vesicle, including the oculogenic transcription factor Pax6 [21] and the presence of Wnt signaling pathways [22], are crucial for epithelial growth. The neural crest gives rise to corneal stromal keratocytes (fibroblasts) and endothelial cells.

**Figure 2:**
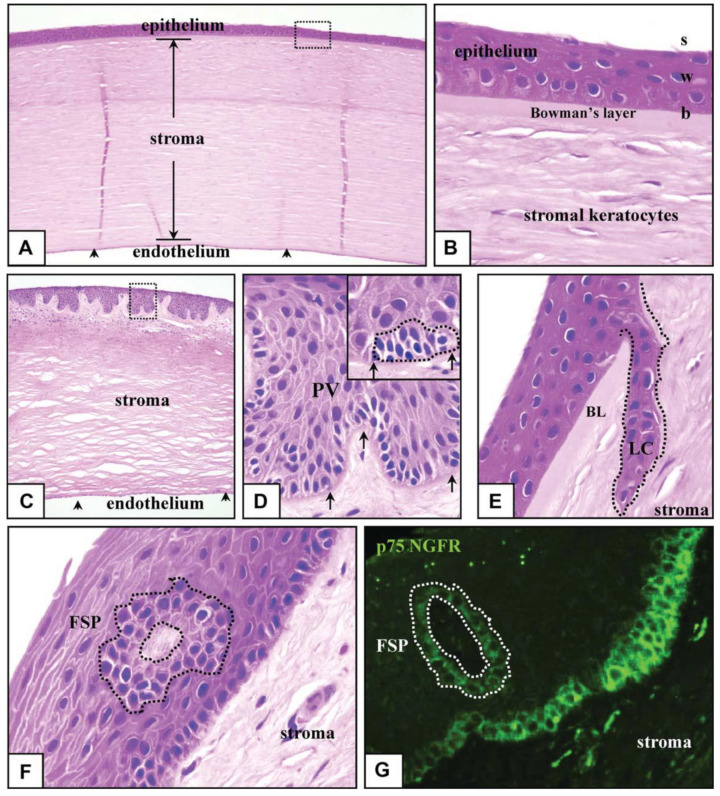
Human corneo-limbus pathologic characteristics. Entire human eye sections were segmented, stained with eosin and hematoxylin (**A**–**F**), as well as for the low-affinity NGFR (p75) (**G**), and visualized using normal (**A**–**F**) or fluorescence (**G**) microscopy. The middle cornea is made up of a multilayered epithelium of squames (s), basal (b) and wing (w) layers, a keratocyte including a monolayer and stroma with complex endothelial cells (**A**,**C**, arrowheads). BL separates the corneal epithelium from the stroma (**B**). The limbus is distinguished by the PV (**C**,**D**), which contains bundles of small stem-like cells (**D**; inset, hatched line). LC (**E**, hatched line) and FSP are two other newly discovered stem cell-harboring structures (**F**,**G**, hatched lines). The limbal membrane is shown by the arrows in (**D**). The boxed region in (**C**) is magnified (**D**). Apart from (**A**,**C**) (100), and (**G**) (400), all pictures were taken with an optical microscope using oil (1000). BL stands for Bowman’s layer, FSP stands for focusing on PV stands, stromal projections, LC stands for limbal crypt and NGFR stands for nerve growth factor receptor for Palisades of Vogt.

The adult conjunctiva is a lamellar tissue consisting of a stratified epithelium, an inner monolayer of the endothelium, with each cell form divided by a specialized membrane and a collagenous substantia propria (stroma) sparsely filled with keratocytes, Bowman’s layer Descemets and anterior membrane. The corneal epithelium is divided into three levels: b (basal), W (wing) and S (squames), as represented in Figure 2A,B. Matrix molecules secreted by b cells may be inserted into the BM (basement membrane) and stroma. W and S cells form from b cells. S cells form lateral close junctions that guard against the environment, while W cells help with wound healing [23]. Constant cyclic repair and shedding, also known as ‘‘self-renewal,” ensures corneal purity and the ability to perform refractive and preventive functions. Forty years ago, in vivo animal experiments repeatedly showed that cells migrate centripetally and circumferentially from the limbus toward the middle cornea through re-epithelialization [24]. Moreover, limbal tissue transplants have been used successfully to treat patients with serious corneal damages [25,26,27,28], providing convincing evidence that the limbus is the reservoir for CESCs. LSCs (limbal stem cells) are found in the Palisades of Vogt’s basal inter-palisade epithelial papillae, as illustrated in Figure 2C,D, and are mostly seen in short chains, as demonstrated in Figure 2D [29,30]. Cells expressing LSC factors can be found in limbus areas rich in crypts and stromal projections [31]. Within the limbus, scientists have discovered crypt-like structures, as shown in Figure 2E, and stromal-like projections, as represented in Figure 2F, consisting of tightly packed b-cells that stain for p75 on limbal epithelial progenitors, as depicted in Figure 2G [32].

## 3. Identity and Location of CESCs (Corneal Epithelial Stem Cells)

For several years, the position of CESCs has been intensively studied, and it is still a very involved and rather contentious field of study. The prevalent and commonly accepted model holds that CESCs are only found at the limbus, which is located at the intersection of the conjunctiva and the cornea. This is supported by data from a number of tests. To begin, epithelial cells in the limbal epithelium’s basal layer feature is especially useful for young, undifferentiated cells, which are consistent with the existence of SCs (stem cells). In particular, epithelial cells in this region lack expression of cytokeratins 3 and 12, that are produced by adult, separated corneal epithelial cells, but maintain expression of cytokeratin 14, that is produced by immature stem or progenitor cells in the basal cell layer of a range of stratified epithelia. Moreover, several cells in the limbus possess putative stem cell receptors. They contain the N isoform of p63, that is represented by progenitor cells or proliferative stem cells in many stratified epithelia, and the transmitter protein ABCG2, which imparts that this is a ‘side-population’ phenotype and is often thought to be a common stem cell indicator [1,3,33]. Some putative stem cell indicators produced by cells in this area contain N-cadherin and Fzd7 [33]. Moreover, the limbal epithelium comprises a higher percentage of quiescent cells that barely differentiate, a characteristic shared by lengthy stem cells in a number of other tissues [34]. While its expression pattern of these indicators is typically compatible with the existence of stem cells, it is significant to mention that a definitive phenotype for ocular epithelial stem cells that corresponds with true stem cell activity has yet to be established.

The most compelling evidence suggesting the existence of stem cells in the limbus is the indication that cells derived from this area can readily produce long-term cell proliferation clones in vitro and can reconstruct the conjunctiva based on transplanting. Evidently, the clinical utilization of stem cells derived from the limbus demonstrates their clinical effectiveness, when they are utilized to repair the conjunctiva in patients who have sustained major damage to the corneal layer as a result of disease or injury [3,35].

**Figure 3:**
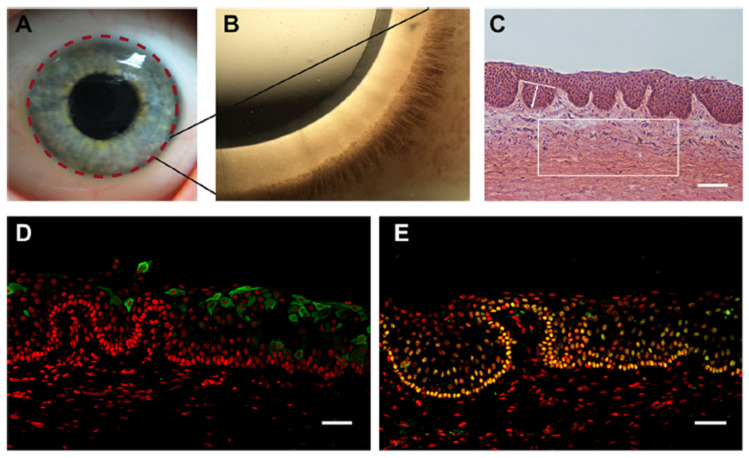
The human limbal stem cell niche. (**A**) Describes the location of the limbus with dashed lines on the human ocular surface. (**B**) Shows a highly pigmented Palisades of Vogt that is visible in the limbus of human. (**C**) Indicates H and E-stained tangential section of the human limbus, showing the LCs (limbal crypts). The box indicates a representative area of 0.1 mm^2^ of the limbal stroma. The white line indicates an example of LC width measurements and arrowed line LC depth measurements. (**D**) CK3- and (**E**) p63a (green)-stained cryosections of human LCs counterstained with PI (red). Scale bars C: 100 µm, D and E: 50 µm [39].

LSCNs (limbal stem cell niches) have been discovered in the limbus, as indicated in Figure 3A, especially in the palisades of Vogt, which are 150-square-meter structures [36]. The Palisades of Vogt have been described as anatomical stromal crypt structures that are particularly noticeable in some intact corneas due to the abundance of melanocytes, which are highly pigmented, as shown in Figure 3B. Separating fixed limbal tissue tangential to the central cornea and staining with H and E revealed stromal protrusions, which establish crypt-like structures that allow for the formation of cell layers in certain areas, as represented in Figure 3C. Longevity, high capacity for self-renewal with a long cell cycle period and short S-phase length, increased potential for error-free proliferation and poor differentiation are all characteristics of SCs. The conjunctival and corneal epithelia, which make up the ocular surface, are made up of two different types of epithelial cells. Even though the two cell phenotypes are anatomically related at the corneoscleral limbus, they are distinct subpopulations. Corneal stem cells are found in the corneoscleral limbus. The Vogt limbal palisades and the inter-palisade rete ridges are thought to be the sources of SC. The limbus microenvironment is thought to be critical in preserving SC stemness. Conjunctival epithelial cells are usually prevented from progressing onto the corneal surface by limbal SCs, which serve as a shield to them. However, under some circumstances, the limbal SCs can be partially or completely depleted, resulting in various degrees of SC deficiency and corneal surface disorders. This can lead to reduced vision and ocular discomfort [37].

A new study discovered evidence of stem cell development on the cornea layer in a host of mammalian mammals, such as the rabbit, rat and pig. SCs strains were removed from many areas of the cornea, including the limbus, except they were found in greater abundance in the peripheral cornea and limbal epithelium. A lineage-tracing procedure also revealed that cells originating from the limbus only belong to the cornea throughout reconstruction and stay inactive throughout homeostasis [38]. As a result of these issues, SCs in the limbus do not substantially contribute to the homeostasis of the corneal epithelium, but they do play an essential regenerative role after damage. 

One potential reason for these results is that CESCs in the limbus are a latent species that only becomes active during wound healing, while CESCs in the corneal epithelium provide the majority of regular homeostatic maintenance. The existence of several stem cell communities is associated with results from other identity tissues, including the bone marrow, epidermis and stomach [40,41,42]. Remarkably, there is substantial evidence in these tissues that various stem cell components lead differently to tissue repair based on the conditions. Dormant colonies of SCs have been found in both the bone marrow and intestine, that only become active throughout wound healing, while a distinct SC niche facilitates the tissue’s homeostatic repair. It is also likely that limbal SCs are a dormant population of SCs that act only under acute conditions, while natural homeostasis is maintained by progenitor cells or another stem cell spread across the eye. More research is required to determine the relative roles of limbal and non-limbal stem cells in corneal layer homeostasis and recovery. Nevertheless, it is apparent that the ocular epithelium includes SCs of exceptional regenerative ability.

## 4. The Corneal Stem Cell Niche

In order to maintain tissue equilibrium, the behavior of stem cells in every environment must be closely controlled. In this regard, the tissue niche or microenvironment, in which stem cells live, is crucial in controlling stem cell important factors when deciding [43]. The SCN (stem cell niche) is a specialized, different anatomical area of a tissue that provides the necessary microenvironmental clues to sustain a cell population capable of satisfying the tissue regeneration requirements of a tissue during any particular time. The crypt core of the small intestine, which contains intestinal epithelial SCs [44], and the bulge area of skin cells, which contains cutaneous epithelial SCs, are also representations of SCNs [40]. A multitude of signals are delivered inside these specialized microenvironments, ensuring proper SC function. The exact molecular and cellular principles by which the niche governs SC behavior, however, are only now being elucidated. Nonetheless, it is clear that a number of niche elements, such as the ECM, vasculature and mesenchymal cells, play a significant role in supplying a variety of signals that affect SC important factors when deciding [43,45], such as aqueous solution metabolic factors, biochemical factors and mechanical cues and also cell-contact-dependent signals [43]. 

Despite the uncertainties regarding the position and identification of CESCs in the corneal crust, all experimentally obtained data suggest that they are strongly enhanced in the limbal epithelium. The limbus is therefore an area of great importance in terms of defining the niche materials that govern CESCs. 

The limbus has some characteristics that differentiate it from the conjunctiva and the cornea on the human ocular surface. Perhaps most dramatically, the stromal tissue in the limbus produces papillae-like invaginations, recognized as the ‘Palisades of Vogt,’ and in between them are limbal epithelial crypts. Inside these crypt systems, a high percentage of basal epithelial cells produce putative SC indicators, such as Fzd7, N-cadherin and ABCG2, reinforcing the idea that the limbus provides a specialized stromal ecosystem designed to support CESCs. Furthermore, the limbal stroma is more vascularized and incorporates different ECM components than the corneal stroma (1 and 2 collagen IV, 2 laminins and vitronectin), and both of these could be essential in CESC maintenance. There seems to be proof that extreme physical contacts between epithelial cells in the limbal crypts and mesenchymal cells in the limbal stroma play a role in SC maintenance [33]. 

The biochemical pathways by which the various elements of the limbal stroma can control the CESC environment need to be investigated further. Nevertheless, limbal stromal cells have been linked to SC repair through the regulation of different biochemical factors. Wnt ligands [22,33,46], that are essential in other SCNs such as cytokines and chemokines such as IL-6 are examples [2], and the intestinal crypt [47]. Specific connections with ECM materials and vasculature, and also cell-contact-dependent mechanisms such as the Notch signaling cascade, could also be significant [33].

The material characteristics of the underlying tissue have been seen to be critical in controlling SC activity. Topography and elasticity, for example, are also used to affect how a cell responds to other microenvironmental signals, including growth factors and/or cytokines [48]. In this respect, SCNs frequently have a specific topography and are made up of complex ECM elements, including one that endows the niche with unique mechanical properties [45,49]. The dome-like structure of the ocular surface is likely to exert distinct impact force at various areas of the tissue, which could favor SC repair at particular positions [38]. Moreover, the limbal stroma’s unique ECM composition can confer a distinct molecular structure. This is supported by evidence that the limbus is slightly tighter than the main cornea [50]. The niche also retains proper physiological requirements for SC repair, such as food supply and oxygen stress, many of which can have a significant impact on SC activity [51].

As a result, distinct limbal stromal features including such ECM structure, vascularization and growth factor function are critical in sustaining a functional population of CESCs. Analyzing how dermal ESCs are controlled by their microenvironment provides some insight into the molecular and cellular mechanisms for which niche elements control CESCs. Biochemical factors naturally produced by mesenchymal cells within the SCN are also used to regulate processes, including SC contemplation and stimulation in this tissue. Development of BMPs (bone morphogenetic proteins) by mesenchymal niche elements, for instance, enhances equanimity of dermal ESCs [52], while expression of fibroblast growth factors (FGFs), TGF and the BMP promoter noggin induces migration and cell growth [53,54]. Functional experiments in mice have also shown that the vasculature is critical in the stimulation of cutaneous ESCs, but the processes are still unknown. Other epithelial materials of the skin were shown to facilitate cell proliferation insertion in quiescent cutaneous ESCs by SHH (Secreting Sonic Hedgehog). Besides that, other materials found in the cutaneous SCN, including such ECM components, peripheral nerves and immune cells, were involved in SC activity regulation [49]. It would be fascinating to see if similar molecular and cellular pathways regulate CESC activity in the cornea. Furthermore, as previously mentioned, the skin contains several SC compartments, each with its own niche. Provided that SC activity has been shown at several positions on the ocular layer, it will be critical to determine if separate niches also exist to sustain this organ. 

### Topography

Different studies have introduced many natural substrates as suitable stem cell carriers for regenerating medicine. Although these studies were relatively successful, there is a strong need to develop alternative synthetic biodegradable cell carriers and increase the cell survival. Therefore, control of cell behavior via the inclusion of microfeatures within biomaterial devices is an emerging area of interest. More precisely, a good amount of research has been conducted by different researchers globally, using various polymeric and synthetic biomaterials for many applications within the human body, which mainly used the electrospinning technique, including breast [55], bone [56,57], nerve [58], dental [59,60], skin [61,62,63], cornea and contact lenses [64,65,66,67,68,69,70], blood vessel [71], ligament [72], diaphragm [73], trachea [74,75], lung [76], cartilage [77], bladder [78] and intestine [79].

To comprehend how biomaterial interface properties including certain stiffness, topography and chemistry will influence cell behavior, scientists should first understand how cells conform to substrates. The layout of a cell’s microenvironment includes stimuli varying from the micro- to the nano-scale, where microscale characteristics are smaller than the cell on its own and result in entire-cell behaviors such as cell coordination with topographical characteristics, including touch guidance [80]. Nevertheless, nanoscale characteristics include a plethora of signals that are many orders of magnitude lower than those provided by the cell [81]. 

Transmembrane and integrin receptors which attach cells to the ECM, that creates an interlayer of proteins deposited on the layer of serum-exposed components [82,83], are usually involved in cell obedience to surfaces. Integrins are heterodimeric proteins with a- and b-subunits that bind to peptide patterns on ECM proteins, such as the aspartic acid (RGD) tripeptide, arginine and glycine [84]. These connections initiate intracellular signaling clusters, most notably G protein activation, which leads to phosphorylation of MLCK (myosin light-chain kinase) via ROCK (rho-associated protein kinase), raising actin–myosin contraction and resulting in integrin classification and cell adhesion configuration [85,86]. Adhesion configuration is dynamic—cells probe their surroundings with unbundled, actin-driven membrane projections known as filopodia. Filopodia are also used to obey touch direction signals as low as 10 nm [87]. Nanoscale projections have been observed at sub-10 nm length scales, demonstrating cellular sensing’s high accuracy. Touch guidance was not seen at this sub-10 nm wavelength, only aspect connections [88]. 

Lithography techniques have been used to produce nanopatterned polymers with regulated size, form, spacing and symmetry in a range of components, and patterns also include nanogrooves [89], nanopits [90,91,92] and nanopillars [93]. Controlling these nano-features provides control over the scale, quantity and distance of adhesions. Large, extreme adhesions (>5 mm long) are seen to be needed for MSC (mesenchymal stem cell) osteogenesis [94]. Intracellular tension is enhanced by producing substrates that facilitate enhanced adhesion size, and this conformational alteration is related to modifications in the cytoskeleton, that can pass tensile (contractile) forces to the nucleus, possibly by cytoskeletal tensegrity [95,96], and raised intracellular tension is related to osteogenesis [94]. Such variations in the nucleus form can have an effect on chromosomal arrangements [97,98,99], and these variations can have an effect on SC phenotype [100].

The topography–protein interface is likely to drive certain improvements in mechano-transductive cell fate, cell adhesion and cytoskeletal organization. Even so, when FN (fibronectin), a main cell-adhesive protein of the ECM, is absorbed through nanopit patterned surfaces, its molecules are adsorbed inside the cells, and it probes these pits with filopodia, resulting in ‘nanoimprinting’ of the pits on the cell membrane, an outcome which was not seen once the layer was not coated in FN [101]. Nanoimprinting is seen to be cell-adhesion-controlled, with adhesion to topographical characteristics resulting in b cell cytoskeleton simulations of the topography. Nanoimprinting is not possible if integrins are blocked [102], showing the role of the ECM in cell reaction to form indirectly. This implies that topography-driven improvements in cell cytoskeleton composition and adhesion are regulated by protein adhesive interfaces, and that cells associate with these interfaces in response to topography [101].

Subsequent spreading and cell adhesion, which regulates scale and form, have an effect on physiological cell functions’ proliferation. Utilizing microcontact-printed ECM areas of declining scale, it has been demonstrated that cell confinement controls have control over proliferation and death, with limited regions that prevent spreading, contributing to cell death [103]. Although, this strategy has been used to restrict MSCs in particular morphologies when regulating intracellular tension and adhesion. MSCs have been unable to form advanced adhesions on ECM forms/islands when they stayed round, which resulted in adipogenic lineage dedication. MSCs were driven to osteogenesis by ECM forms/islands/scales that enabled mature adhesion formation, spreading and facilitated actin–myosin contractility [85,86]. The cytoskeleton’s actomyosin tension leads to this structural regulation, that is physiologically related to adhesion formation regulated at the nanoscale by variations in the cytoplasm. It is also worth noting that altering the lipid structure of the cytoplasm will influence intracellular signaling and thus SCF (stem cell fate) [104].

To decouple adhesion conditions for cell spreading, topographical RGD-coupled substrates were utilized. Integrins bind to the actin cytoskeleton as they ligate. The integrins are joined together by activating G-proteins, which causes actin–myosin contraction, contributing to the forming of mature adhesions composed of several integrins. It has been shown, utilizing nanocolloid samples with one RGD motif bonded to every colloid, that integrin grouping will happen when RGD is at a density of <70 nm apart; beyond this density, integrins cannot cluster together again and shape matured adhesions [105,106]. It was discovered that utilizing electron beam lithography techniques to build clusters of RGD within 70 nm of one another, the small groups dispersed beyond obtaining distance, while tetramers of obtained integrins were needed for full cell spreading, such as functional adhesions [107]. 

**Figure 4:**
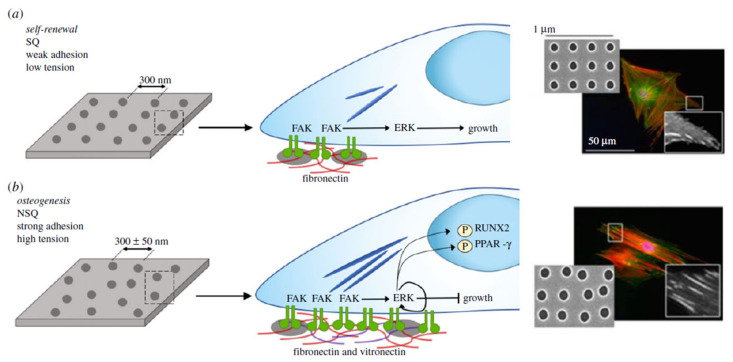
Controlling MSC adhesion for osteogenesis and self-renewal by topography. (**a**) Self-renewing MSCs bind weakly compared to osteo-committed cells, leading to a reduction of integrin-mediated signaling via focal adhesion kinase (FAK), while retaining levels of extracellular signal-regulated kinase (ERK1/2) to promote growth but not separation. (**b**) MSCs inducing osteogenesis need greater adhesions: enhanced FAK activation raises ERK1/2 activation to lineage commitment levels, raising intracellular stress, activating Runt-related transcription factor 2 (RUNX2), a central regulator of osteogenesis, although subsequently inactivating adipogenic controller peroxisome proliferator-activated receptor gamma (PPAR-y). Fluorescent photographs display a significant improvement in adhesion length on near square (NSQ) surfaces relative to square (SQ). Adjusted from the source [92].

This topographical regulation upon how cells bind to surfaces has been utilized to regulate even osteogenic differentiation or self-renewal of MSCs, despite the fact that topographies can be strikingly similar. In a SQ (square) lattice, for instance, an electron beam lithographically determined structure that allows out-of-niche self-renewal is composed of holes with a radius of 60 nm, depth of 50 nm and center spacing of 150 nm [91]. MSC destiny is changed to osteogenesis by adding only +25 nm deviation from the center location and shifting the layer to NSQ (near square) [90]. As previously mentioned, adhesion length varies greatly between surfaces, with MSCs generating smaller adhesions and experiencing less intracellular strain on SQ relative to NSQ [92], as illustrated in Figure 4. It is worth noting that cells on the NSQ surface switch from FN to vitronectin as their endogenous ECM output [108]. Vitronectin has been linked to increased cell capacity to make connections in the ECM, which indicates that if enough integrins are clustered in two close positions, intracellular linker proteins, including vinculin, will span the distance throughout the multiple places, even if no integrin ligands are visible; this is known as merging, and vitronectin is a more successful merging protein than FN [109]. 

## 5. The Limbal Stem Cell Niche

The limbal stem cell niche, located at the anatomic boundary of the cornea and the conjunctiva [39,110,111], generates a microenvironment that aids in the growth and repair of their signals, resident cells and ECM, that identify a SCN [110,112]. Due to the occurrence of melanin pigmentation, the corneoscleral limbus has an identifiable protective atmosphere with thick protection, vascularization and innervation from possible light destruction [20,113,114]. The limbal palisade and corneal transformation areas tend to regulate cell proliferation sensing, creating a distinct microenvironment for progenitor cells and CESC. Though elements of the SC membrane in the dorsal limbus could provide external stimuli that lead to stemness repair [30,43,115,116,117], elements of the delayed progenitor cell membrane in the anterior limbus could control the phenotypic variations required to regenerate the restoring corneal epithelium [30].

While the presence of the limbal niche is acknowledged, particular details of its 3D architecture remain unknown [118]. Research of the construction of the limbal crypts and the corneal limbus has been performed utilizing various methods, with various mechanisms discovered [31,112,113,114,119,120,121,122]. Goldberg and Bron (1982) and Townsend (1991) utilized a pit light to observe the corneal limbus and identified the Vogt palisades as a set of circular patterns aligned fibrovascular ribs clustered along the superior and inferior corneoscleral limbus, differentiated by inter-palisade epithelial varied forms. They discovered a large variation of the palisade region from one human to the next and within the same eye, as well as a large diversity of the form of inter-palisade epithelial crypts and palisades, such as palisade branching, radially directed rectangular and/or circular or oval shapes, or connectivity to create a trabecular structure [113,114]. Even so, due to the high variability, Goldberg and Bron (1982) concluded that the limbal palisade design is as unique as a fingerprint [113]. Limbal crypt design has been investigated utilizing histopathology [9,112,121,122], OCT (optical coherence tomography) [119,123], confocal fluorescence microscopy [31,124], electron microscopy [31,112,122] and in vivo confocal reflectance microscopy [31,120,122,125]. Utilizing imaging and electron microscopy, Shortt et al. defined the presence of both LCs between the FSPs and palisades of Vogt at the ocular layer of the limbus, expanding in a finger-shaped from the palisades and thus becoming circular/oval in the facial image processing zone of the limbus [31]. Histology was used by Dua et al. (2005) and Shanmuganathan et al. (2007) to record the presence of wider, fewer frequent crypt systems, known as LECs (limbal epithelial crypts), that included oblique, radial and circumferential interlinking elements and descend from the epithelial cells to beneath the corneal layer [112,122]. Imaging refractive microscopy, such as the HRT II with the Rostock device (Heidelberg, Germany), can conduct non-invasive visualization of the limbal area in in vitro and/or in vivo tissues [120], although its limited field scale avoids observing the entirety of the limbal area in-depth in a specific purchase, and cross-section data is not accessible. Electron microscopy and histology are both harmful, including tissue slicing, fixation and staining. Confocal microscopy and OCT imaging of the 3D configuration of the limbus in static individual corneoscleral rims [119] showed a mixture of the configurations, such as a number of inter-palisade and palisade structures with inner- and outer-individual heterogeneity, as well as patterns that could correlate to FSPs, LCs and LECs. 

### Microenvironment Structure 

Present advances in computing technology, as well as the use of molecular and cellular research methods, have resulted in a better view of the limbal microenvironment [7,30,118,126,127,128]. The limbal niche includes ridges identified as the palisades of Vogt, which lead to epithelial and stromal undulations. In particular, the epithelium reaches further into the limbal region and is distinguished by alternating stromal regions that occur as lines on clinical inspection (palisades). The LESCs are present in the b cells of the limbal epithelium in these regions, that are also known anatomical structures, as LECs [112,126,129]; however, LESCs were never identified in any LEC, and specific distribution structures of LESCs have been reported in various persons [6,112,118]. These microenvironments have distinct gene expression and ECM protein profiles that are optimal for the installation and repair of LESCs [30,126,127]. In addition, stromal (mesenchymal) cells [130], melanocytes [131], immune cells [127], vascular cells [129] and nerve cells [132] are found in the limbal niche. 

Due to their role in LESC control, MSCs have gained a lot of attention in recent decades. Mesenchymal CD105- and CD90-positive cells are also used to interact closely with LESCs [7,118,128,133]. IVCM (in vivo confocal microscopy) revealed clusters of hyper-reflective MSCs in the anterior limbal niche stroma adjacent to the basal epithelial cells, where LESCs are found [133]. MSCs are also used to interact with LESCs through a variety of chemical materials and signal transduction pathways, including IL-6/STAT3 [2], aquaporin-1 and vimentin [2,134], chondroitin sulfate (6C3 motif) [128], SDF-1/CXCR4 [135] and BMP/Wnt [136]. Paracrine growth factor secretion and intercellular touch [137,138], as well as similar impacts on cytokine and growth factor expression [139], are additional mechanisms of communication. In vitro, vimentin-positive shape cell infiltration derived from human limbal tissue has been seen to regenerate the limbal niche and recellularize decellularized human corneas [140]. 

**Figure 5:**
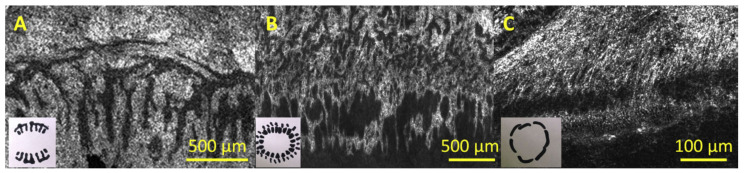
Interspecies variation in limbal morphology: human, pig and mouse. En face FFOCM (full-field optical coherence microscopy) pictures of (**A**) human, (**B**) pig and (**C**) mouse limbus, with inset diagrams of geometry of crypt formation about 360° of the eye. FFOCM pictures are oriented with the sclera on top and the cornea on the right. Photos have been measured to represent an identical area of every cornea (though differently sized due to different eye sizes) [6].

Figure 5 illustrates the comparisons between human, pig and mouse LSCNs. In pig eyes, as seen in Figure 5B, the palisade region was centered on the corneal part of the limbus rather than the ocular part. Palisades were fine, and inter-palisade crypts were broad and circular, with a consistent circular direction and a trabecular design connected to various small invaginations under the cornea. Both eyes had equal thickness (50 mm), diameter (90 mm), form (oval) and orientation (radial) distribution. The limbal zone was 1.5 mm in width. The palisades of Vogt were not found in fresh mouse ocular surface, and the limbal zone included just endothelial cells roughly circling the cornea, as represented in Figure 5C [6].

The design of the limbus remained intact after 1 month in organ culture, despite the fact that the epithelial tissue had softened, and layer cells had become deformed.

## 6. The Limbus and Other Stem Cell Niches

In the last five decades, it was suggested that a SCN offers a distinct and sufficient microenvironment for multipotential operation and to promote self-renewal [17]. Niches are 3D (three-dimensional) SC-sheltering, finely ordered, dynamic structural elements that are typically found at tissue boundaries, intersections or areas; for instance, endo-ectocervical, cornea-limbal and esophagogastric [18]. The differential clues that determine SC homeostatic or stimulation programs are assumed to be provided by molecular crosstalk from neighboring cells and reversible signals from the subsequent vasculature or ECM-sequestered intermediaries specific to the microenvironment. Chemical and physical signals between the 3D spaces’ cells and matrix glycoproteins they shape enable for intermolecular forces that are essential for controlling SC activity. The detection and classification of tissue niches has identified a constellation of materials; nevertheless, the processes governing how niches are formed and preserved to serve SC roles are only now being established [19]. Furthermore, new methods for marking SC in vivo have made it easier to identify and characterize SC niches in mammalian cells [1,2,19,141]. 

The hair follicle has appeared for one of the most thoroughly researched adult SC templates. Multipotent HFSCs (hair follicle stem cells) that regenerate skin, hair and sebaceous glands exist in the hair follicle bulge, an area of the basal part sheath. Previous studies that took to the benefits of bulge cells’ weak cycling allowed them to be identified and isolated as label-retaining progenitors [34,142]. When such cells were grafted into hairless mice, they developed huge colonies in situ and unchanged hair follicles. The identification of molecular techniques to help classify HFSCs has greatly enhanced scientists’ and researcher’s knowledge of their anatomy and biology, such as their adhesion to ECM proteins and the molecules related to cell regulation.

The pathways involved in regulating adult HSCs (hematopoietic stem cells), such as the HFSC niche, are widely undefined. HSCs are a type of bone marrow-derived cell that can self-renew or differentiate into several types of cells. HSCs that are dormant cross the inner layer of bone filled with osteoblasts [143]. When HSCs reach maturity, they cross paths with the stromal cells around them and continue to propagate. Multiple studies containing osteoblast-ablated mice and mice bioengineered to enhance osteoblast number have led to one regulatory hypothesis, which indicates that HSCs maintain their quiescent features owing to their ability to bind to osteoblasts via N-cadherin-mediated adherent intersections [143,144]. Another study found that HSCs produce calcium-sensing receptors, and that HSCs lacking these receptors did not locate to the endosteal niche and did not act regularly after grafts [145]. This emphasizes the significance of the ionic mineral composition of bone and its structure in HSC preservation within the niche [143,144]. Moreover, osteoblasts have been shown to produce angiopoietin 1, which associates with a tyrosine kinase inhibitor on quiescent HSCs, improving their adhesive capacity and providing an ideal condition for hematopoiesis [146]. 

The cornea’s clarity sets it apart from other SC-containing organs. In addition, owing to its shallow anatomical position, the limbus seems to be the only SCN that can be easily observed utilizing noninvasive small-hole and in situ imaging techniques. The widely held belief is that unipotent LSCs within the basal cells of the limbus preserve the ocular epithelial cells through natural cellular proliferation and after injury [22,23,147]. Studies demonstrating the movement route of pigmented cells from the limbal region [20] provide insights as to the position of cells with regenerative potential. Following that, Cotsarelis et al. [34] offered compelling evidence of slow-cycling, label-retaining, stem-like cells at the limbus that gradually shed their radiolabel as they progressed into the middle of the cornea. Latest chimera experiments have shown a radial striping form of TACs (transient amplifying cells) flowing from the limbus into the middle of the cornea [34,148].

## 7. Reconstruction of the Limbal Stem Cell Niche

A variety of methods have been used for regenerating the limbal epithelial cells (LECs) and preserving the niche. Past research has shown that in serious cases of LSCD, simply administering LESCs cannot be used for long-term corneal layer regeneration [149,150,151,152,153,154]. ESCs in particular are likely to be damaged if grafted to a hostile corneal layer ecosystem, where a stable SCN cannot be re-established. Systemic inflammation and dysfunction of adhesion molecules and the ECM are the main reasons for limbal niche disruption after insults. As a result, techniques for reconstructing the LSCN are concentrated on preserving proper function and reducing inflammation of adhesion molecules and ECM [12]. Among these methods are the use of scaffolds/matrices, mesenchymal stem cells and hemoderivatives. 

### 7.1. Bio-Active Extracellular Matrix for Limbal Niche Replacement

A tissue regeneration ECM can almost certainly be used in a competitive limbal niche reconstruction technique.

#### Fabricating Bio-Active ECMs for Niche Reconstruction

The limbal niche is a 3D system made of an incorporated ECM. As a result, supplementing a tissue regeneration-constructed ECM may be a viable technique for restoring the limbal niche’s role. The protocols for developing bioactive ECMs commonly depend on the use of purification structural proteins, including human corneas or collagen or the decellularization of animal proteins. Limbal crypts were created utilizing type I collagen and sheet molding. This genetic engineering of crypts promoted the growth and physiology of human LESCs, while also supplying the required framework for integration at the crypt’s surface [155]. Similarly, an innovative method for synthesizing cell-laden ocular structures is to use 3D printing with a bio-ink combination of laminin, collagen and elastin [156,157].

Other methods for generating a bio-active ECM have been analyzed: human corneas and decellularization of porcine [158,159]. These procedures focus on the use of ribonucleases, detergents and osmotic solutions to eliminate all cellular functions and minimize antigenicity. The bioavailability of decellularized corneas has been demonstrated by developing CECs on prepared scaffolds and even grafts in animal studies [158,159,160]. Furthermore, several clinical trials have looked at the outcomes of decellularized porcine cornea implantation in cases of ocular ulceration [161,162]. Since this technique is most often used in circumstances involving stromal substitution with safe epithelium, its application to LSCD could be minimal. As a result, digesting the decellularized corneas and creating a hydrogel has been suggested as a possible protocol for fabricating a bio-active ECM. The hydrogel that was created received adequate support for in situ hybridization of ocular stromal cells [163,164]. Making a bio-active ECM hydrogel from decellularized corneas may be a viable method for re-creating the limbal niche. Overall, the definition should include a bio-active ECM that contains protein molecules and also healing agents.

### 7.2. Cell-Based Approaches for Restoring the Limbal Niche in Mesenchymal Stem Cells

In recent decades, MSCs have received a lot of recognition for their potential use in regenerating the limbal niche and cornea layer. Fridenestein and colleagues were the first to separate MSCs from bone marrow lesions in 1968. They discovered that certain adherent epithelium cells can regenerate bone tissue. Additional research has shown that these cells have the ability to repair compromised tissues [165]. The immunomodulatory attributes of MSCs are one of the essential features that make them ideal for use in organ transplantation and patients with autoimmune diseases [166]. In 3D culture structures, MSCs can also generate ECM [167,168]. The ISCT (International Society for Cellular Therapy) has determined that human MSCs must meet the following basic requirements [169]:

1. Plastic conformity.
2. Separation of osteocytes, adipocytes and chondrocytes.
3. Negative expression of CD14, CD34, CD45 and HLA-DR, and positive expression of CD105, CD73 and CD90.

A variety of experiments have focused on the application of MSCs from different origins in mouse models of corneal layer diseases, such as corneal transplantation [170], chemical burns [171,172], dry eye syndrome [173] and limbal stem cell defects [174]. MSCs limbus [174,175], bone marrow stromal (BM-MSCs) [176], adipose tissue (AD-MSCs) [177], HAM (human amniotic membrane) [178] or omentum [179] have all been examined in the restoration of ocular layers. The paracrine influence of BM-MSCs tends to increase LECs’ proliferation and operation in situ [180]. For instance, following native BM-MSCs, thermal ocular injury moves from bone marrow to inflamed cornea as a consequence of enhanced SDF-1 (stromal cell-derived factor-1) and substance-P levels in compromised cornea and blood plasma. Pursuing the localization of BM-MSCs on the compromised cornea, enhanced concentrations of anti-inflammatory cytokines such as TGF (transforming growth factor) and IL-1Ra (Interleukin-1Ra) will contribute to important ocular epithelial layer reconstruction [181]. It has also been demonstrated that MSCs bone marrow stromal cells can reduce the production of cytokines (TNF and IFN) and immunomodulatory components induced by damaged ocular epithelial cells [182]. BM-MSCs could also contain advantageous soluble factors, such as EGF, to help rebuild the limbal microenvironment [183]. It has also been demonstrated that BM-MSCs applied to the chemically damaged corneal epithelium cells will minimize the mediators of lipid peroxidation and oxidative stress, leading to a reduction in the proportion of pro-inflammatory cytokines and apoptotic cells, such as IL-1, IL-2 and IFN, as well as reduced ocular neovascularization [171,184,185].

## 8. Production of BLCs (Bioengineered Limbal Crypts) in RAFT (Real Architecture for 3D Tissue) Constructs 

**Figure 6:**
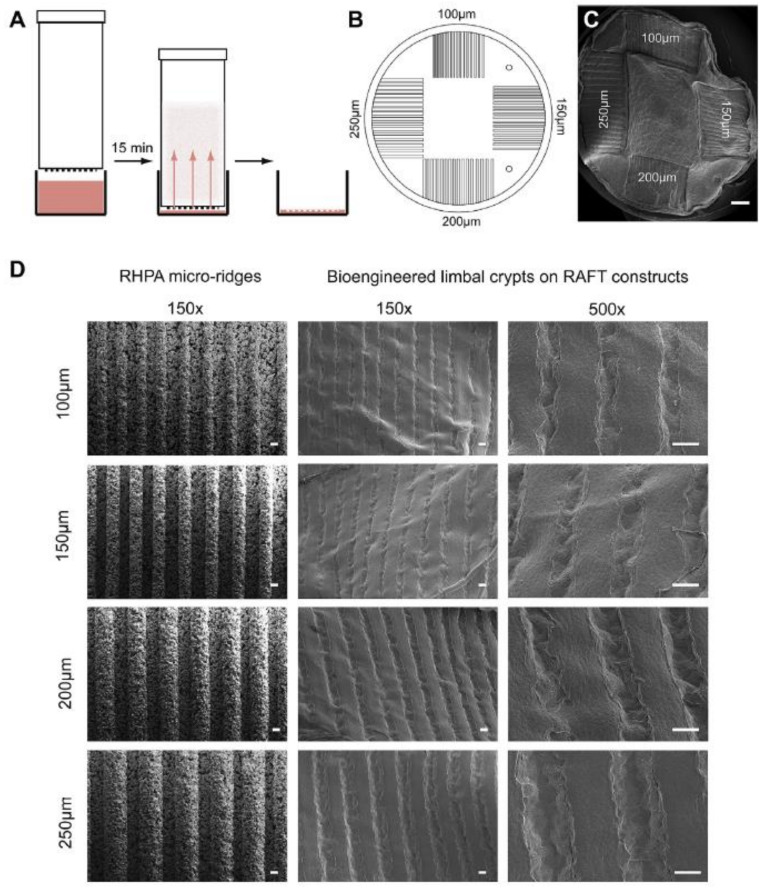
Production of BLC-containing RAFT constructs using RHPAs. (**A**) Shows a schematic of the RAFT process using RHPAs which are placed on top of a collagen hydrogel in a 24-well plate and incubated for 15 min. This will allow wicking of liquid from the hydrogel. RHPAs are then removed, and the RAFT construct remains at the bottom of the well. (**B**) Shows a schematic pattern of topography of micro-ridges on the base of RHPAs whiles. (**C**) Shows SEM images of a RAFT construct showing four different topologies on the same surface. (**D**) Shows the SEM images of the protruding micro-ridges of variable depth on the RHPA surface and corresponding BLCs produced in the surface of the RAFT constructs. Scale bars, C: 1 mm, D: 100 µm [39].

HPAs (hydrophilic porous absorbers) suitable for use in a traditional 24-well tissue culture plate are used in the RAFT process. HPAs are mounted on the surface of collagen hydrogels and incubated on a plate heater set to 37 °C for only 15 min. The liquid is wicked from the hydrogel and absorbed by the HPAs during this period. The HPAs are then removed, leaving only thin RAFT constructs attached to the well plate’s foundation, ready to receive a cell suspension on the surface, as illustrated in Figure 6A. It is possible to build micro-scale crypts on the surface of RAFT constructs using this method, which mimic the 3D physical structures of human LCs. A mold tool is designed to manufacture several different sized protruding micro-ridges on the HPA surface to establish the correct requirements for the topology on the base of the HPAs. Each ridged HPA (RHPA) had four sets of micro-ridges with equal widths and depths of 100, 150, 200 or 250 m, surrounding a flat central region, as demonstrated in Figure 6B. BLCs of various depths and widths are seen on a single RAFT build, depending on the dimensions of the initial micro-ridges on the RHPA, as represented in Figure 6C. The porous material characteristics, as well as the uniformity in size of the micro-ridges, are visible in SEM (scanning electron microscopy) images of RHPAs, as shown in Figure 6D [39].

### 8.1. Characterization of BLCs

**Figure 7:**
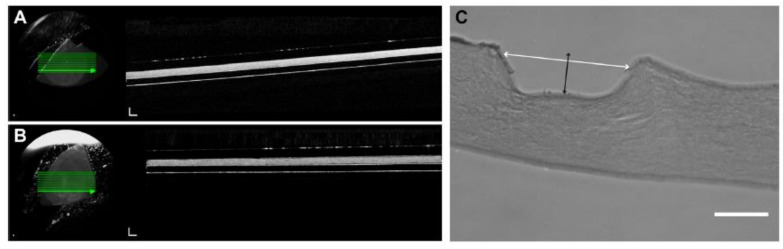
BLCs in RAFT constructs. (**A**) Shows a representation of an OCT image of an unfixed RAFT construct produced using an HPA. (**B**) Shows a representation of an OCT image of an unfixed RAFT construct produced using a RHPA. (**C**) Shows a representation of the H and E-stained section of a BLC on the surface of a RAFT construct. The white arrow shows width and the black arrow shows the depth measurements. Scale bars, C: 50 µm [39].

The addition of the micro-ridges in the RHPAs is tested using OCT imaging to see how the overall thickness of the RAFT constructs is affected. The HPA constructs had an average thickness of 152.5 ± 9.19 µm after 3 weeks in culture, as shown in Figure 7A, while the RHPA constructs had an average thickness of 165.8 ± 9.61 µm, as represented in Figure 7B. This distinction is not considered to be statistically important (*p* > 0.05). Since it is difficult to distinguish between cells and collagen on OCT pictures, and the crypts are cell-filled, the BLCs are not apparent in these images. Transverse parts of paraffin-embedded RAFT constructs are used to calculate the width and depth of crypts to determine the dimensions of the generated BLCs, as indicated in Figure 7C [39].

### 8.2. Cell-Filled BLCs

**Figure 8:**
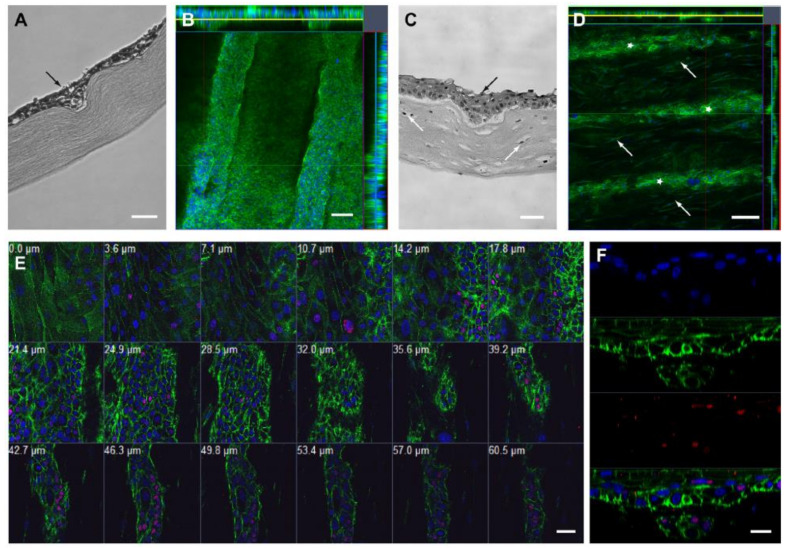
Cell-filled BLCs. (**A**) Shows an H and E-stained paraffin-embedded section of HCE-T cell-filled BLCs (black arrow) on RAFT construct. (**B**) Represents an orthogonal confocal image of HCE-T cells in BLCs stained with phalloidin (green) and DAPI (blue). Yellow line indicates Z-stack position on *X*-axis and blue line on *Y*-axis. (**C**) Shows an H and E-stained paraffin-embedded section of HLE cell-filled BLCs (black arrow) on the surface of HLF (white arrow) containing RAFT constructs. (**D**) Is a representation of an orthogonal confocal image of HLE cells in crypts (white stars) and HLF cells (white arrows) within the RAFT construct, both stained with phalloidin (green) and DAPI (blue). Yellow line indicates Z-stack position on *X*-axis and blue line on *Y*-axis. (**E**) Shows gallery view of a series of confocal Z-stack images showing HLE cell-filled BLCs and HLF cells within the RAFT construct stained with p63a (red), phalloidin (green) and DAPI (blue), with the depth from the epithelial surface indicated in mm. (**F**) Shows the confocal line scan image of the HLE cell-filled BLCs stained with p63a (red), phalloidin (green) and DAPI (blue). Scale bars: A, C: 50 µm, B, G–I: 100 µm, D: 200 µm, E: 40 µm, F: 20 µm [39].

Cell seeding experiments on RAFT constructs with BLCs made with RHPAs are optimized using HCE-T cells. Cells developed an epithelium of approximately 3–4 cell layers on the flat regions of the RAFT build after 2 weeks in culture, according to histological sections. Layering increased as cells filled the BLCs in areas with crypt topology, creating multilayers 5–7 cells deep, as represented in Figure 8A. The multilayering of cells as they filled the length of crypts is highlighted by confocal Z-stack images and orthogonal views, as shown in Figure 8B [39]. 

On the flat surface of RAFT constructs containing HLFs, HLE (human limbal epithelial) cells developed a healthy 3–4-cell multi-layered epithelium. HLEs filled the crypts where BLCs are present, creating a multi-layered epithelium of 6–7 cells in some areas, as seen in Figure 8C. Cells filled the length of crypts, and HLFs could be seen in near proximity to the cell-filled crypts in the underlying bioengineered collagen stroma, according to confocal Z-stack images and orthogonal views, as illustrated in Figure 8D. As the optical parts shifted from superficial epithelium (0.0 µm from the surface) to the base of a BLC (60.5 µm), as demonstrated in Figure 8E, a sequence of confocal Z-stack images clearly highlighted the transition in cell size and morphology. The presence of large, squamous epithelial cells with a low N/C ratio and no p63 expression is a characteristic of the superficial epithelium. The cell-filled crypts with HLFs in proximity inside the RAFT build became apparent as the series progressed. The expression of p63, a putative stem cell marker, increased in tandem with the N/C ratio of cells at this stage, and high p63 expression is noted in cells lining the base of BLCs, with 79.2 ± 9.5 percent of basal crypt cells expressing p63. The presence of a multi-layered epithelium and expression of p63 in the basal layers was revealed by a confocal line scan that optically sectioned a cell-filled crypt, as represented in Figure 8F [39].

## 9. Artificial Stem Cell Niches for Self-Renewal

Processes for MSC separation are being better understood, thanks to the use of biomaterial interfaces. Acknowledging MSC self-renewal in situ, on the other hand, is becoming increasingly critical. MSCs have predominantly been utilized in regenerative medicine techniques, but they are indeed being examined for their tumor-homing abilities for drug delivery [186,187] and as anti-inflammatories to attenuate transplantation disease [188,189,190]. To support this, MSCs must be separated and extended in situ, which is difficult due to normal cell culture plastic’s lack of self-renewal ability.

**Figure 9:**
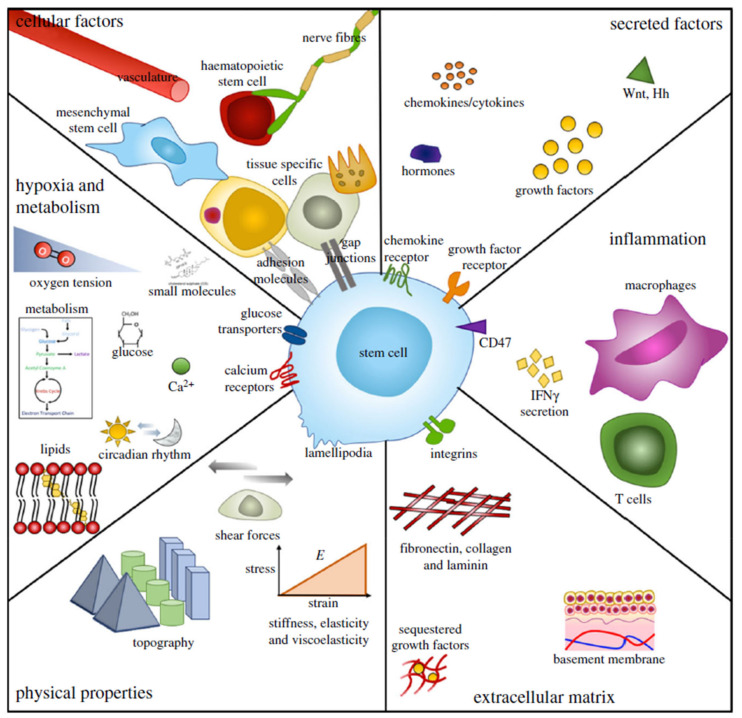
Characteristics of SCs and their niches. Niches are multi-factorial and dynamic microenvironments that are distinct and exclusive to function, but many of their primary parameters are shared. Physical and complex influences, including cell–cell interactions and heterologous cellular functions, secreted and soluble or membrane bound factors, immunological activity and reaction, ECM protein elements and properties, physical architectural parameters, oxygen stress and metabolic regulation, are all included [196].

As illustrated in Figure 9, the niche is a diverse setting. It is worth noting that in specific molecular drug research, the push for higher sensitivity, excessively simplistic cell models that do not reconstruct cell niches, and testing on animals in non-human models has fueled a competitiveness crisis in which a vast number of chemical products are being advanced, many of which fail in clinical trials. This homeostasis between separation and self-renewal, proliferation and quiescence, is strictly regulated in situ by a variety of niche-specific variables, as represented in Figure 9. In situ SC self-renewal is being studied by modification of the biomaterial properties described earlier. As previously stated, nanotopographies with very good formation have been used, and an arranged square design contributes to retained multipotency of MSC indicators across large culture cycles [91]. Muscular stem cell tissue regeneration capability has been preserved in culture microenvironments that resemble the natural toughness of muscle [191], and basic chemical modulation of glass slides has also been utilized to provide hydroxyl groups that preserve the MSC phenotype [192]. It is worth noting that toughness modulation of MSC self-renewal is still elusive. Nevertheless, it has been shown that environments with relatively homogenous toughness do not help cell proliferation or heterogeneous situations. Unorganized structures of matrix dynamics have been seen to cause prolonged expression, cytoskeletal destruction and reduced cell spreading of MSC-related marker proteins utilizing biodegradability polymers [193]. Furthermore, nanoparticle-based methods have been utilized to keep MSCs alive. MSCs are magnetically glided into spheroids inside collagen type I gels with the aid of nanostructured materials. They remained dormant and expressed niche/MSC indicators including stro-1 and nestin in this 3D niche. Furthermore, by utilizing a basic wound-healing model in which the spheroid-niches were placed over monolayers of various traits (chondrocyte, fibroblast and osteoblast) that were then scratched, the cells responded to the tissue regeneration requirement with differentiation, engraftment into the necessary phenotype [194] and migration. It is worth noting that when intact, unscratched monolayers have been utilized, the MSCs in the niches stayed dormant. This is an important finding in regenerative healing, but other than that, dormant in 3D in situ niche.

Acknowledging the molecular mechanisms, adult SC self-renewal, especially of MSC, has been minimal. MSC self-renewal necessitates an intermediate adhesion state that inhibits differentiation while allowing for long-term growth in situ. Many biomaterial approaches have illustrated that osteogenesis of MSCs involves large adhesions that sustain high intracellular tension [90,92], while adipogenesis opposes this, appearing when adhesion is small and tension is minimal [85,86]. Situations favoring MSC self-renewal are located in the middle of these two fates. Nevertheless, since these situations still favor fibroblast development, performing this in culture has proven difficult [81,195]. 

Since in situ SCNs are complicated, combinatorial biotechnology techniques are being developed to enable the reconstruction and deconstruction of these multilayered mechanisms. One method to enlighten on SCN processes is to simplify the mechanism. Microarray operating systems that enable screening of the impacts of variable degrees of numerous microenvironmental signals on SCF have been produced to accomplish this. At the single-cell level, robotic locating technologies can introduce and analyze a combinations of protein cues, including niche interaction ligands, ECM components and other signaling proteins [197,198]. One experiment in cell culture for human neural precursors on protein-printed arrays discovered that cells stayed indistinct only when activated by two morphogens in conjunction, Notch and Wnt [199]. This method has also been utilized to analyze ligands results in the transformation of mammalian cells to myoepithelial or luminal epithelial fates [200]. More currently, Roch et al. introduced ligand elements of the bone marrow niche and defined candidates essential for HSC repair, identified to HSCs, and utilizing single-cell examination, and they were able to identify gene expression signatures of HSCs as they separated into MPPs (multipotent progenitors) [201]. 

PEGDA (polyethylene glycol diacrylate) hydrogels are promising scaffolds for providing 3D models in aqueous environments for tissue regeneration. However, due to the non-adhesive existence of PEGDA chains, PEGDA hydrogels usually exhibit minimal or no intrinsic biological activity. Due to the critical properties such as good biocompatibility, non-immunogenicity and resistance to protein adsorption, PEGDA has been a popular form of hydrophilic polymer for biomedical applications, such as surface modification, bioconjugation, drug delivery and tissue engineering. PEGDA can be crosslinked to form a hydrogel that imitates the ECM condition for cell encapsulation [202]. 

PEGDA is a photocurable synthetic biocompatible polymer that can be crosslinked by irradiation when a suitable photo-initiator is present. PEGDA is commonly used in tissue engineering applications as well as the development of microenvironments for SC research [203,204].

**Figure 10:**
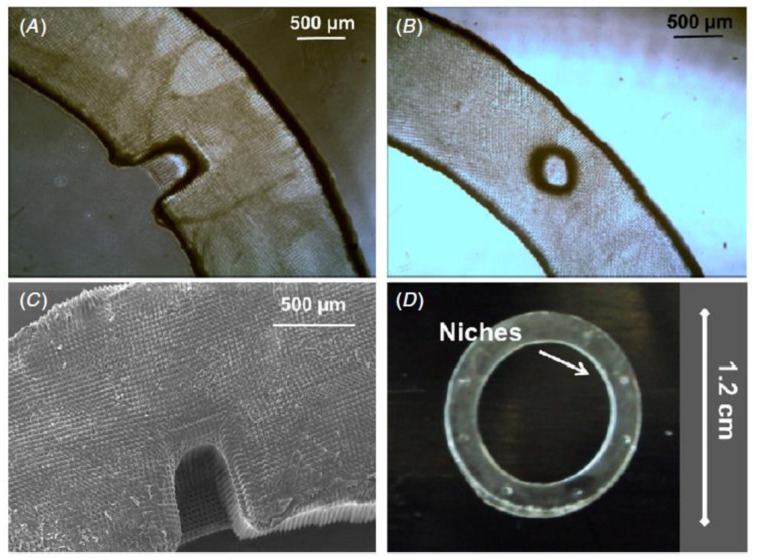
Phase contrast microscopy and scanning electron microscopy were used to investigate the structure of the PEGDA rings. (**A**) Describes the optical micrographs of the PEGDA outer ring with horseshoe morphology. (**B**) Describes the circular morphology of the micrographs of PEGDA. (**C**) Describes a SEM micrography of the PEGDA outer ring with horseshoe niches. (**D**) Shows a PEGDA outer ring of diameter 1.2 cm with well-defined artificial micro-pockets of diameter around 300 µm [206].

**Figure 11:**
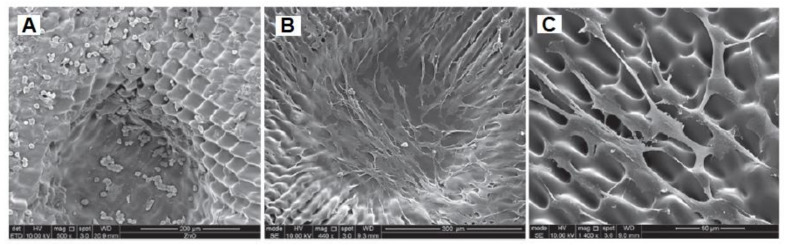
(**A**) Shows a washed sample of the SEM images of cells in the short term. (**B**) Shows a washed sample of the SEM images of cells in the long term. (**C**) Shows an SEM high-magnification image of well-attached RLF on PEGDA surface [206].

Niches are anatomically established locations with complex structural, physiochemical and metabolic cues. They provide the position and microenvironment for cells to have the ability to self-renew for a lifetime [205]. When attempting to establish artificial SCNs, current theories indicate that several important features are required: physical security for epithelial cells, secure attachment of these cells to a suitable ECM in these niches, and finally, the presence of stromal cells directly adjacent to the epithelial cells, as shown in Figure 10. The depth of niches varies, as shown in SEM images represented in Figure 10C and Figure 11A,B [206].

The MTT assay is used to assess the effect of the PEGDA outer rings on cell viability. Cells are cultured for various periods of time on PEGDA rings that had been washed with PBS (sodium phosphate buffer). SEM is used to examine RLF (rabbit limbal fibroblasts) morphology before and after washing. Cells grown on short-washed samples produced round-shaped cells, suggesting weak attachment, which was consistent with MTT findings indicating cytotoxicity, as shown in Figure 11A. Cells with a stable and more natural appearance with the characteristic elongated fibroblastic morphology were helped by samples washed for longer periods, as represented in Figure 11B. As seen in the high-magnification SEM image, the cells were well-attached to the structures, even though they followed the microfabricated patterned surface, as indicated in Figure 11C [206].

## 10. Conclusions and Perspectives

In conclusion, this article concentrated on the CESCN and CLSCN with ECM in the microenvironment using different approaches. There is presently no conclusive marker for illuminating LSCs or distinguishing them from their earlier TAC offspring. Although, integrins seem to be suitable markers because their expression on the cellular membrane provides a tethering point for evaluating, isolating and enhancing cells. When combined with suitable ECM factors, advances in culture conditions and existing cell-based treatments for individuals with LSCD may be achieved. In addition, since SCs of certain self-renewing epithelia share molecular structures, findings from corneal studies can help researchers to improve treatment options for other body organs. By mimicking both the close interaction of neighboring niche cells and the geometry of the 3D microenvironment, RAFT (real architecture for 3D tissue) offers an ideal in vitro system for studying the behavior of LESC.

The layout of the LSCN tends to be related to LSC, individual behavior and eye orientation, for instance, nocturnal or diurnal, interior or lateral side and appearance or absence of brows. Besides that, clone generation in humans was closely associated with the amount of limbal crypts, suggesting that limbal crypts serve as a niche for adult LSCs. FFOCM imaging can aid in determining the sensitivity of the limbal crypts for selective biopsy for tissue culture in the development of synthetic bioengineered corneas.

Long-term restoration of LESC activity requires rebuilding of the LSCN. Recent tissue regeneration therapies include the use of genetic or artificial scaffolds, as well as growth factors, hemoderivatives or cytokines. Similarly, MSCs, with their active immunomodulatory effects and capacity to generate trophic and ECM factors to help LESCs, are presently a potential candidate for cell-based treatment for recovering the limbal niche. This research also includes a prototype system and an ex vivo ocular system for studying the behavior of cultured SCs in these synthetic niches, the 3D form of which can be changed as needed and the layer of which could be adjusted with a variety of ECM proteins. 

CESCs, like all SCs, are extremely sensitive to their microenvironment, and improper microenvironmental clues may result in their degradation and/or impaired operation. As a consequence, an improved comprehension of how the niche regulates CESCs and how niche elements shift through illness or injury has the potential to lead to enhanced preventive options for a range of corneal layer conditions Finally, the discovery of molecular techniques that explicitly classify CESCs would be a major step forward, allowing researchers to investigate the relationships between CESCs and their microenvironment in a more precise and systematic manner.

## Data Availability

Not applicable.
